# Association of occupational lead exposure with erythrocytic parameters and nitric oxide–related metabolites: a case-control study

**DOI:** 10.55730/1300-0144.6364

**Published:** 2026-04-12

**Authors:** Melih Gaffar GÖZÜKARA, Servet Birgin İRİTAŞ, Deniz ÖZKAN VARDAR, Lütfiye TUTKUN, Özlem İRİTAŞ, Serdar DENİZ, Vugar Ali TÜRKSOY, Engin TUTKUN

**Affiliations:** 1 Department of Public Health, Faculty of Medicine, Ankara Yıldırım Beyazıt University, Ankara, Turkiye; 2 Department of Forensic Medicine, Faculty of Medicine, Ankara Yıldırım Beyazıt University, Ankara, Turkiye; 3 Pharmacy Services Program, Vocational School of Health Services, Lokman Hekim University, Ankara, Turkiye; 4 The Association of Industrial Toxicology and Occupational Hygiene, Ankara, Turkiye; 5 Republic of Türkiye Ministry of Agriculture and Forestry, Department of Foreign Relations, Ankara, Turkiye; 6 Department of Public Health, Faculty of Medicine, Malatya Turgut Özal University, Malatya, Turkiye; 7 Department of Public Health, Faculty of Medicine, Yozgat Bozok University, Yozgat, Turkiye; 8 Tez Medikal Institute of Occupational Health and Safety, Ankara, Turkiye

**Keywords:** Lead exposure, asymmetric dimethylarginine, symmetric dimethylarginine, erythrocyte indices, nitric oxide metabolism

## Abstract

**Background/aim:**

Occupational lead (Pb) exposure remains a significant public health concern due to its persistent presence in industrial environments and its cumulative toxic effects. Although the hematological and neurological consequences of Pb exposure are well documented, early vascular effects and the mechanisms associated with potential endothelial impairment remain unclear. This study aimed to investigate the associations between Pb exposure, erythrocytic parameters, and nitric oxide (NO)–related metabolites (asymmetric dimethylarginine [ADMA], symmetric dimethylarginine [SDMA], arginine, homoarginine, and citrulline) to identify early biomarkers that may suggest impairment in NO-related pathways.

**Materials and methods:**

This case-control study included a strictly selected cohort of 125 occupationally Pb-exposed workers and 125 healthy controls without known occupational exposure. To minimize confounding, all participants were strict nonsmokers and nondrinkers with no history of chronic disease. Blood Pb levels, hematological parameters, and metabolic biomarkers, including arginine, ADMA, SDMA, homoarginine, and citrulline, were analyzed using standardized laboratory methods. Associations between Pb levels, erythrocyte indices, and amino acid–related metabolites were evaluated using correlation analysis.

**Results:**

Blood Pb concentrations were significantly higher in the Pb-exposed group. Pb-exposed individuals had significantly lower hemoglobin (Hb) levels. While ADMA levels did not differ significantly between groups, SDMA levels were higher and citrulline levels were lower in the exposed workers. Ferritin levels were lower, whereas transferrin and total iron-binding capacity levels were higher in the exposed group. In multivariable analyses adjusted for age and body mass index, blood Pb levels were associated with higher SDMA levels and lower Hb, citrulline, and ferritin levels.

**Conclusion:**

Occupational Pb exposure was associated with alterations in erythrocytic indices and NO-related metabolic markers, which may be consistent with impairment in NO-related pathways. However, because of the case-control design and the absence of direct endothelial function measurements, these findings should be interpreted as associative rather than causal.

## 1. Introduction

Lead (Pb) remains one of the most widely distributed and biologically harmful heavy metals despite substantial reductions in global exposure over recent decades. As a nonessential metal with no physiological role, Pb exerts toxic effects across multiple organ systems and is recognized as a major public health and occupational hazard [1–4]. Both acute and chronic exposure have been associated with neurocognitive impairment, nephrotoxicity, reproductive dysfunction, hematologic disturbances, and increased cardiovascular morbidity and mortality [5,6].

A central driver of Pb toxicity is oxidative stress, which is characterized by excessive generation of reactive oxygen species, depletion of antioxidant reserves, and disruption of redox-sensitive pathways [3]. Oxidative stress contributes to endothelial nitric oxide synthase (eNOS) inhibition and reduces nitric oxide (NO) bioavailability, promoting vasoconstriction, inflammation, and endothelial dysfunction. In parallel, Pb disrupts heme synthesis through inhibition of δ-aminolevulinic acid dehydratase and ferrochelatase, increases erythrocyte membrane fragility, and accelerates red blood cell (RBC) turnover, leading to microcytic hypochromic anemia and altered erythrocyte morphology [1,7]. Because erythrocytes play a regulatory role in vascular NO signaling by modulating NO transport and scavenging, Pb-induced hematologic alterations may further contribute to endothelial injury.

Beyond classical hematotoxicity, attention has shifted toward the molecular determinants of endothelial dysfunction, particularly the L-arginine/NO pathway. Asymmetric dimethylarginine (ADMA) is an endogenous inhibitor of eNOS, while symmetric dimethylarginine (SDMA) and L-homoarginine also influence NO availability and endothelial function. The L-arginine/ADMA ratio serves as a more sensitive indicator of NO bioavailability than ADMA concentrations alone [8]. Elevated ADMA and reduced arginine/ADMA ratios have been linked to hypertension, atherosclerosis, renal disease, and adverse cardiovascular outcomes. However, despite biological plausibility, the relationship between occupational Pb exposure, methylated arginine profiles, and endothelial function remains insufficiently understood, and the existing evidence is inconsistent [9,10].

While research has extensively characterized Pb neurotoxicity and general systemic effects, fewer studies have examined its early biochemical signatures within the erythrocyte–NO axis. Understanding whether alterations in methylated arginine metabolism accompany hematologic changes in exposed workers may provide mechanistic insights into Pb-related vascular risk. The identification of such biomarkers is particularly relevant for occupational surveillance, where subclinical endothelial impairment may precede clinically apparent cardiovascular disease.

In this context, the associations between occupational Pb exposure and erythrocytic parameters and methylated arginine metabolism were investigated in a carefully selected cohort of asymptomatic workers and controls. RBC indices and plasma concentrations of ADMA, SDMA, L-homoarginine, L-arginine, and citrulline were quantified, and their relationships with blood Pb levels were assessed. Rather than directly measuring endothelial dysfunction, the focus was placed on biochemical markers related to NO metabolism that may provide indirect insight into impairment in NO-related pathways. In addition, correlations between blood Pb levels and biochemical parameters were assessed, and the influence of exposure duration (1–5 versus ≥6 years) on biomarker profiles was examined.

Previous studies examining the vascular effects of Pb exposure are often confounded by lifestyle factors such as smoking, alcohol consumption, and underlying cardiovascular conditions, which independently impair NO bioavailability. By utilizing a strictly selected cohort devoid of these major confounders, the specific associative role of occupational Pb exposure was aimed to be isolated. The aim was to delineate early hematologic and endothelial alterations associated with Pb exposure, thereby contributing mechanistic insight and supporting biomonitoring strategies in occupational toxicology.

## 2. Materials and methods

### 2.1. Study design and population

A case-control study involving a total of 250 participants was conducted. The case group consisted of 125 adult individuals with occupational Pb exposure, and 125 age- and sex-matched individuals without known Pb exposure served as controls. All participants were otherwise healthy workers recruited from an industrial region in which Pb is commonly used (e.g., battery manufacturing and metal processing facilities). To minimize potential confounding factors—particularly those known to influence NO metabolism and hematologic indices—and to ensure homogeneity between groups, stringent selection criteria were applied. Inclusion criteria required participants to be male, aged 25–60 years, and to have at least 1 year of employment in their current position. To isolate the specific effects of occupational Pb exposure, all enrolled subjects in both the exposed and control groups were strictly nonsmokers and nondrinkers. Furthermore, individuals were excluded if they had any history or clinical evidence of chronic cardiovascular, renal, hepatic, metabolic, respiratory, or endocrine diseases (including ischemic heart disease, hypertension, diabetes mellitus, thyroid disorders, and chronic kidney disease). Additional exclusion criteria included the presence of active infections or inflammatory conditions, malignancy, prostate pathology, and recent exposure to other heavy metals, organic solvents, or environmental pollutants outside the workplace. Finally, subjects using multivitamin and mineral supplements (e.g., zinc, selenium) or any medications known to influence vascular or hormonal regulation (e.g., statins, antihypertensive agents, corticosteroids, or hormone replacement therapies) were also excluded from the study. Informed consent was obtained from all participants, and the study was approved by the institutional ethics committee (Ankara Bilkent City Hospital Medical Research Ethics Board; decision no. TABED 1-24-1113; dated 12 March 2025) and was conducted in accordance with the Declaration of Helsinki.

Basic demographic data (age, sex, and body mass index [BMI]) and occupational history were recorded for all subjects. The Pb-exposed workers were further subdivided into two subgroups based on exposure duration: 1–5 years (n = 53) and ≥6 years (n = 72). This stratification was chosen to assess the impact of prolonged chronic exposure. The cutoff (5 years) approximately corresponded to the median exposure duration in the sample and was consistent with prior classifications in occupational studies.

### 2.2. Statistical analysis

Data were analyzed using IBM SPSS Statistics for Windows (version 26.0; IBM Corp, Armonk, NY, USA). All continuous variables were tested for normality using the Kolmogorov–Smirnov test. Continuous variables were presented as mean ± standard deviation (SD), and categorical variables (e.g., sex) were expressed as frequencies and percentages.

Group comparisons were performed using appropriate statistical tests. The Pb-exposed and control groups were compared using the independent-samples t-test for continuous variables. To evaluate the effect of exposure duration within the Pb-exposed cohort, independent t-tests were used to compare the 1–5 year and ≥6-year subgroups. Parameters showing statistically significant differences are marked in the tables to highlight meaningful effects.

Pearson correlation analysis was performed to assess the relationships between blood Pb levels and various biological parameters (e.g., hemoglobin (Hb), ferritin, ADMA). Correlation coefficients (r) and their significance (p value) were calculated, with p < 0.05 considered statistically significant. In all analyses, a two-tailed p < 0.05 was used as the threshold for statistical significance. To further explore independent associations, multivariable linear regression analyses were performed for selected dependent variables (SDMA, Hb, citrulline, arginine, platelet count, and ferritin). Blood Pb levels, age, and BMI were included as independent variables in all models using the enter method. Multicollinearity was assessed using variance inflation factor (VIF) values, with VIF < 5 considered acceptable. Model fit was evaluated using R^2^ and analysis of variance (ANOVA) statistics. Standardized (β) and unstandardized (B) coefficients with 95% confidence intervals were reported. A p < 0.05 was considered statistically significant.

### 2.3. Sample collection and laboratory analyses

Demographic data for the study and control groups were obtained from occupational histories. Whole blood samples were analyzed using the Cell-Dyn Emerald system (Abbott Diagnostics, Abbott Park, IL, USA) to determine hematological parameters. Whole blood samples collected in EDTA tubes were gently vortexed for homogenization before analysis, and 9 μL of blood from each sample was used to determine basic parameters, including erythrocytes (RBC), Hb, hematocrit (HCT), platelets, and white blood cells, as well as the percentages of neutrophils, lymphocytes, monocytes, eosinophils, and basophils. Control samples were used to verify the accuracy of the measurements.

Pb concentrations in blood samples were analyzed using an Agilent 7700 inductively coupled plasma mass spectrometry (ICP-MS) system (Agilent Technologies, Santa Clara, CA, USA). Blood samples (1 mL) were transferred to a high-temperature-resistant microwave digestion system. All samples were digested using a microwave digestion system (Start D; Milestone, Sorisole, Italy). Briefly, 10 mL of nitric acid (65% HNO3; Suprapur; Merck KGaA, Darmstadt, Germany) was added to the samples, acid digestion was performed in the microwave system, and the samples were transferred to 15 mL polypropylene tubes (Isolab, Wertheim, Germany) with screw caps. The total volume was adjusted to 10 mL with ultrapure water [11]. The operating parameters were set as follows: radiofrequency power, 1550 W; nebulizer gas flow, 0.97 L/min; plasma gas flow, 0.9 L/min; nebulizer pressure, 2.98 bar; dwell time, 0.01 ms; and spray chamber temperature, 3.8 °C. The sampler probe was washed between injections by rinsing with ultrapure water for 30 s, followed by washing with 2% HNO_3_ for 45 s, and finally rinsing with ultrapure water for 45 s. After the washing steps, the instrument automatically processed the next sample. A 7-point (0.1–100 ppb) calibration curve was constructed and optimized for blood Pb level analysis. A minimum correlation coefficient (R) of 0.9994 was obtained for the Pb calibration curve. The limit of detection for Pb was determined based on the standard deviation of the response and the slope of the calibration curve [11,12].

The analytical method was validated using certified reference material (CRM) (Whole Blood Level-2; Sero AS, Billingstad, Norway). CRM refers to highly accurate reference materials certified for one or more specific properties and plays a crucial role in ensuring analytical accuracy control while providing reliable and precise results in the analysis of Pb in whole blood samples. Such materials are widely used for method validation and optimization across various applications, particularly in toxicology. The selected CRM consists of more than 95% human blood without the addition of preservatives or stabilizers, thereby closely mimicking natural human blood conditions. This composition enhances the validity of analytical results by minimizing potential matrix effects. Comprehensive product documentation is available, providing certified analytical values for more than 60 elements, with Pb as a primary analyte. These values are independently verified and traceable to international reference materials, ensuring high standards of accuracy and reliability in analytical measurements.

Consequently, CRM serves as an essential tool for researchers and professionals requiring precise and dependable data. To ensure the accuracy and reliability of the analytical results, five replicate measurements were performed for both standard and sample analyses. In the validation studies, CRM samples were analyzed five times per day over 10 consecutive days. Intraday and interday precision assessments were conducted using standard reference materials, with precision determined based on the standard deviation of replicate measurements as a quality control measure. This approach was adopted to minimize variability and to achieve a relative standard deviation of less than 5%. For the Pb method validation study, the coefficient of variation for the Whole Blood Level-2 CRM was 2.19%.

ADMA, SDMA, arginine, homoarginine, and citrulline levels in plasma samples were analyzed using a Shimadzu LC-20AD liquid chromatography system (Shimadzu Corporation, Kyoto, Japan). The system was coupled to an API 3200 triple quadrupole mass spectrometer (Applied Biosystems/MDS SCIEX, Concord, ON, Canada) operating in electrospray ionization positive mode using a Phenomenex Luna C18 column (Phenomenex, Torrance, CA, USA) [13,14]. Blood samples were centrifuged at 3000 rpm for 10 min. Plasma aliquots were stored at −20 °C until analysis. Calibration curves consisting of seven concentration points (0.1–500 μmol/L) were generated for ADMA, SDMA, arginine, homoarginine, and citrulline measurements.

## 3. Results

### 3.1. Comparison of Pb-exposed and control groups

A total of 250 participants were evaluated (125 Pb-exposed and 125 controls). The mean age was similar between the groups (p = 0.299), and BMI did not differ significantly (p = 0.555). Blood Pb levels were markedly higher in the exposed group (31.51 ± 19.41 μg/dL) compared with controls (1.71 ± 0.87 μg/dL) (p < 0.001). In the amino acid and methylated arginine analyses, arginine concentrations did not differ significantly (p = 0.158). Homoarginine was significantly higher in Pb-exposed workers (p = 0.002), whereas citrulline levels were significantly lower (p < 0.001). ADMA levels did not differ between the groups (p = 0.150), whereas SDMA levels were significantly higher in the exposed group (p < 0.001). The arginine/ADMA ratio was significantly lower in the exposed group (p = 0.006), whereas the SDMA/ADMA ratio did not differ significantly (p = 0.826).

Hematologic parameters showed significantly lower Hb, HCT, mean corpuscular volume (MCV), mean corpuscular hemoglobin (MCH), and mean corpuscular hemoglobin concentration (MCHC) values in the Pb-exposed group (all p < 0.001). Platelet count and red cell distribution width (RDW) did not differ significantly (p = 0.273 and p = 0.718, respectively).

For iron metabolism, ferritin levels were significantly lower in the exposed group (p < 0.001), while serum iron levels did not differ (p = 0.473). Transferrin and total iron-binding capacity (TIBC) were significantly higher in Pb-exposed workers (both p < 0.001). Table 1 summarizes the differences in demographic characteristics, hematologic indices, iron metabolism markers, and nitric oxide–related amino acid parameters between Pb-exposed workers and healthy controls.

### 3.2. Comparison according to duration of Pb exposure

Among the Pb-exposed group, 53 individuals had short-term exposure (1–5 years), and 72 had long-term exposure (≥6 years). There were no significant differences in age (p = 0.369) or BMI (p = 0.780) between the exposure-duration groups. No statistically significant differences were observed between the short- and long-term exposure groups for arginine (p = 0.395), homoarginine (p = 0.677), citrulline (p = 0.578), the arginine/ADMA ratio (p = 0.881), the SDMA/ADMA ratio (p = 0.888), HCT (p = 0.170), platelet count (p = 0.813), MCV (p = 0.052), MCH (p = 0.240), MCHC (p = 0.660), RDW (p = 0.512), ferritin (p = 0.107), transferrin (p = 0.540), or TIBC (p = 0.525). However, long-term exposure was associated with higher ADMA and SDMA levels (p = 0.022 and p = 0.008, respectively), as well as lower serum iron (p = 0.005) and Hb levels (p = 0.016). Table 2 summarizes hematologic, iron metabolism, and nitric oxide–related biomarker findings in Pb-exposed workers stratified by short-term versus long-term occupational exposure.

### 3.3. Correlations between blood Pb levels and biomarkers

**Figure 1:**
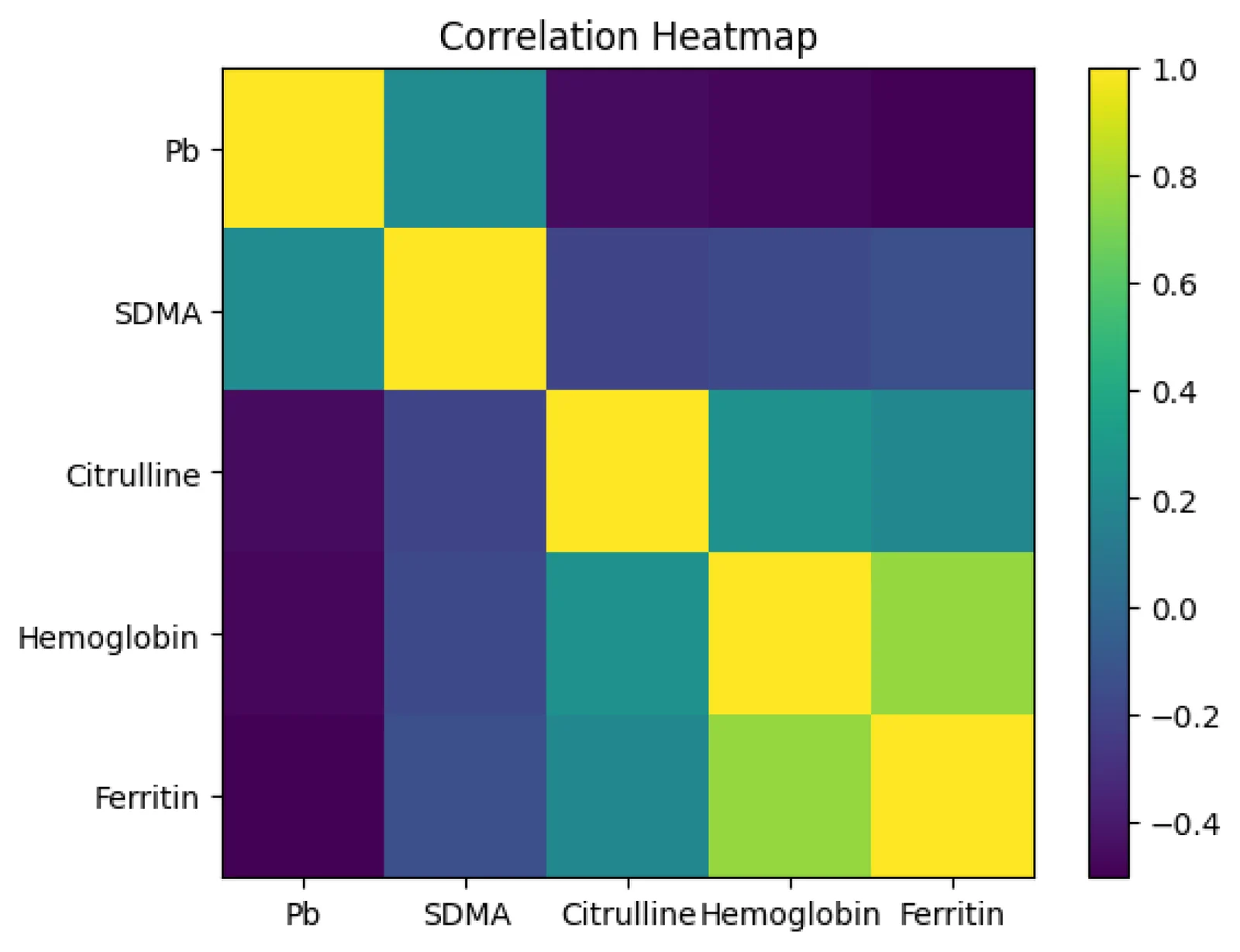
Correlation heatmap of selected biomarkers. The heatmap illustrates Pearson correlation coefficients among blood Pb levels, nitric oxide–related metabolites (SDMA, citrulline), and hematological parameters (Hb, ferritin). Warmer colors indicate positive correlations, whereas cooler colors indicate negative correlations.

Blood Pb levels showed significant negative correlations with Hb (r = −0.476), ferritin (r = −0.503), MCV (r = −0.465), and MCH (r = −0.532), as well as positive correlations with SDMA (r = 0.224), transferrin (r = 0.370), and TIBC (r = 0.355) (all p < 0.01). These relationships are shown in Table 3 and are visually summarized in Figure 1.

### 3.4. Multivariable regression analyses

**Figure 2:**
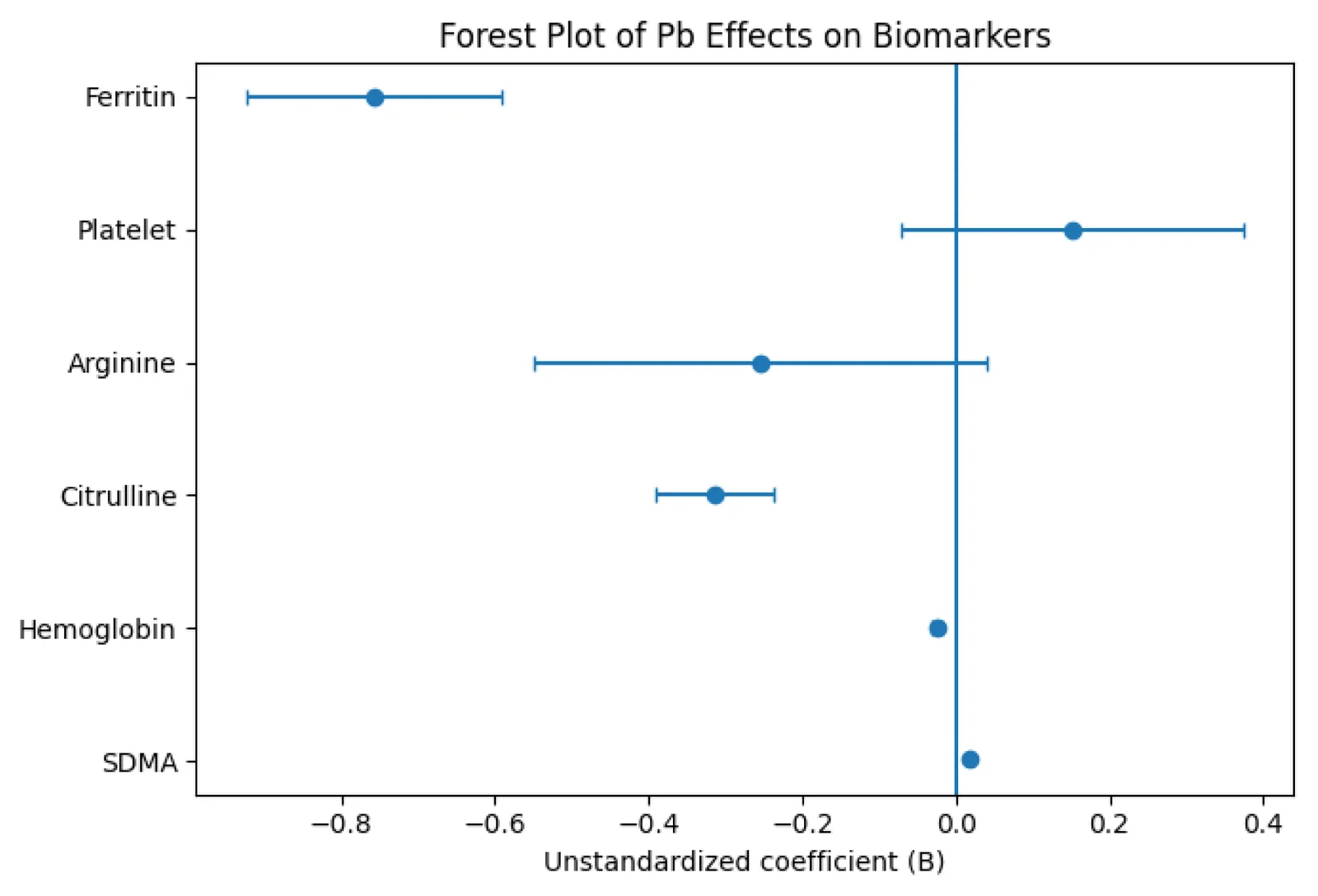
Forest plot of the association between blood Pb levels and selected biomarkers. The plot presents unstandardized regression coefficients (B) and 95% confidence intervals from multivariable linear regression models adjusted for age and body mass index. The vertical reference line indicates no association (B = 0).

Multivariable linear regression analyses showed that blood Pb levels were positively associated with SDMA (β = 0.220, 95% CI: 0.000–0.001, p = 0.001) and inversely associated with hemoglobin (β = −0.464, 95% CI: −0.031 to −0.019, p < 0.001), citrulline (β = −0.453, 95% CI: −0.391 to −0.236, p < 0.001), and ferritin (β = −0.498, 95% CI: −0.924 to −0.591, p < 0.001) after adjustment for age and body mass index (Table 4). No significant independent associations were observed for arginine or platelet count. Age and BMI were not significant predictors in any of the models. Detailed regression coefficients and confidence intervals are presented in Table 4. These associations are further illustrated in Figure 2.

## 4. Discussion

The associations between occupational Pb exposure, hematological parameters, and NO-related metabolites were evaluated in a carefully selected cohort of healthy workers. The findings suggest that Pb exposure is associated with alterations in erythropoiesis, iron metabolism, and selected NO-related metabolic markers. While these observations are biologically plausible and consistent with previous literature, they should be interpreted as associative rather than causal due to the case-control design of the study.

Consistent with well-established mechanisms of Pb toxicity, Pb-exposed individuals showed lower Hb, HCT, MCV, and MCH levels, supporting an adverse effect on erythropoiesis. Pb is known to inhibit key enzymes in heme synthesis, including δ-aminolevulinic acid dehydratase and ferrochelatase, and to increase erythrocyte fragility and turnover [1,7]. The observed reductions in erythrocytic indices are therefore in line with prior experimental and epidemiological findings [15]. In addition, the decrease in ferritin levels and the increase in transferrin and TIBC are consistent with a pattern suggestive of absolute iron deficiency, as previously reported in Pb-exposed populations [1,5,16,17]. These findings support the interpretation that Pb exposure may disrupt iron homeostasis and erythrocyte production, even in the absence of overt clinical disease.

Beyond hematologic effects, alterations in NO-related metabolites were also identified. Citrulline levels were significantly lower in the exposed group, which may be consistent with reduced NO production, given that citrulline is generated as a byproduct of NO synthesis [18,19]. In contrast, SDMA levels were higher in Pb-exposed individuals. SDMA has been associated with reduced renal clearance and may also interfere with L-arginine transport, thereby indirectly influencing NO bioavailability. Although none of the participants had clinically apparent renal disease, the observed increase in SDMA may reflect early or subclinical alterations in renal handling or methylarginine metabolism, as suggested in previous studies [20,21]. However, these interpretations remain indirect, as renal function was not specifically evaluated using sensitive biomarkers.

ADMA levels did not differ significantly between the groups, despite other changes suggesting altered NO metabolism. This finding may be compatible with the so-called “ADMA paradox,” in which NO bioavailability is reduced without a corresponding increase in circulating ADMA levels [14]. In such cases, factors such as reduced substrate availability, altered methylarginine handling, or increased oxidative degradation of NO may play a more prominent role than ADMA concentrations alone [8,22]. In the present study, the observed reduction in the arginine/ADMA ratio and the lower citrulline levels may represent more informative indicators of alterations in NO-related pathways than ADMA levels themselves.

The multivariable regression analyses further support these observations by demonstrating that blood Pb levels were independently associated with higher SDMA levels and lower Hb, citrulline, and ferritin levels after adjustment for age and BMI. These findings may suggest that the observed associations are not fully explained by these covariates, although residual confounding cannot be excluded. The consistency of these associations across multiple biologically related parameters strengthens the interpretation that Pb exposure may be associated with concurrent alterations in hematologic and NO-related pathways.

The absence of a significant independent association between Pb and arginine in the regression models suggests that changes in the arginine/ADMA ratio may be driven more by downstream metabolic alterations than by direct effects on circulating arginine levels. This pattern is consistent with previous observations indicating that NO bioavailability may be influenced by multiple interacting factors, including substrate availability, enzymatic activity, and oxidative stress [8,22].

From a broader perspective, the findings of this study are consistent with emerging evidence that even moderate levels of Pb exposure may be associated with subclinical biological changes relevant to cardiovascular risk [23]. However, given the indirect nature of the biomarkers assessed and the lack of direct functional measurements, these results should be interpreted as hypothesis-generating rather than definitive evidence of endothelial dysfunction. From a public health perspective, the present findings may have implications for the monitoring of occupational exposure. Although the observed biomarker alterations cannot be interpreted as evidence of clinical disease, they may reflect early biochemical changes occurring at exposure levels commonly encountered in industrial settings. These results may therefore support the importance of continued surveillance and preventive strategies in Pb-exposed populations. However, given the case-control design and the indirect nature of the biomarkers assessed, further longitudinal and mechanistic studies are required before conclusions can be drawn regarding clinical or regulatory significance.

The study has several strengths, including the use of a strictly selected population of healthy, nonsmoking, nondrinking individuals without chronic disease, which reduces the influence of major confounders known to affect NO metabolism and hematological parameters. At the same time, several limitations should be acknowledged. The case-control design precludes causal inference. Endothelial function was not directly assessed using functional vascular measurements, and therefore the findings reflect indirect biochemical patterns rather than direct evidence of endothelial dysfunction. Although major confounders were controlled through the study design, residual confounding related to dietary factors, inflammatory status, or unmeasured environmental exposures cannot be excluded. In addition, background environmental exposure in the control group cannot be completely ruled out, although blood Pb levels were substantially lower than those in the exposed group. Finally, the absence of longitudinal follow-up limits the interpretation of the temporal progression and clinical relevance of these findings.

## 5. Conclusion

Occupational Pb exposure was associated with alterations in erythrocytic indices, iron metabolism, and selected NO-related metabolites in this cohort of asymptomatic workers. These findings may be consistent with early biochemical changes affecting both hematologic and NO-related pathways. Further prospective studies incorporating direct measures of endothelial function are needed to clarify the clinical significance and the underlying mechanisms of these associations.

## Figures and Tables

**Table 1:** Hematological and biochemical parameters in Pb-exposed and control groups.

Parameter (with reference ranges)	Pb-Exposed (n = 125)	Control (n = 125)	p-value
	Mean ± SD		
**Age (years)**	39.81 ± 8.11	40.99 ± 8.50	0.299
**Blood Pb (μg/dL)**	31.51 ± 19.41	1.71 ± 0.87	**<0.001**
**BMI (kg/m** **^2^** **)**	28.72 ± 3.66	28.47 ± 3.07	0.555
**Arginine (μmol/L)**	79.25 ± 64.84	87.77 ± 17.21	0.158
**Homoarginine (μmol/L)**	2.67 ± 1.34	2.22 ± 0.83	**0.002**
**Citrulline (μmol/L)**	8.91 ± 7.38	26.33 ± 13.71	**<0.001**
**ADMA (μmol/L)**	0.23 ± 0.16	0.20 ± 0.14	0.150
**SDMA (μmol/L)**	0.26 ± 0.08	0.23 ± 0.04	**<0.001**
**Arginine/ADMA ratio**	396.4 ± 329.5	486.4 ± 141.7	**0.006**
**SDMA/ADMA ratio**	1.26 ± 0.47	1.25 ± 0.34	0.826
**Hb (g/dL) (13.5–17.2)**	13.50 ± 0.77	14.79 ± 1.01	**<0.001**
**HCT (%) (39.5–50.5)**	43.11 ± 2.19	45.15 ± 2.55	**<0.001**
**Platelet count (10** **^3^** **/μL) (150–400)**	227.9 ± 38.7	222.9 ± 32.5	0.273
**MCV (fL) (80–99)**	88.52 ± 2.86	92.26 ± 3.60	**<0.001**
**MCH (pg) (27–33.5)**	29.80 ± 1.27	32.09 ± 1.54	**<0.001**
**MCHC (g/dL) (31.5–36)**	33.39 ± 0.87	34.37 ± 1.24	**<0.001**
**RDW (%) (11.8–14.5)**	14.46 ± 0.68	14.82 ± 11.12	0.718
**Ferritin (μg/L) (22–322)**	50.17 ± 25.57	80.44 ± 28.24	**<0.001**
**Serum iron (μg/dL) (65–175)**	86.06 ± 24.41	88.14 ± 21.22	0.473
**Transferrin (mg/dL) (200–360)**	362.86 ± 90.45	266.02 ± 99.13	**<0.001**
**TIBC (μg/dL) (240–450)**	334.82 ± 72.84	249.94 ± 85.31	**<0.001**

Data are presented as mean ± SD. Statistical significance values were obtained using independent t-tests comparing the case and control groups. Bold values indicate p < 0.05. All participants in both groups were nonsmokers, nondrinkers, and free of chronic cardiovascular or metabolic disease.

**Table 2:** Comparison of hematological and methylated arginine parameters according to exposure duration.

Parameter	Short-term (1–5 years) (n = 53)	Long-term (≥ 6 years) (n = 72)	p-value
	Mean ± SD		
**Age (years)**	39.15 ± 8.51	40.46 ± 7.63	0.369
**Blood Pb (μg/dL)**	28.03 ± 11.44	34.08 ± 23.37	0.085
**BMI (kg/m** **^2^** **)**	28.83 ± 3.52	28.64 ± 3.77	0.780
**Arginine (μmol/L)**	73.48 ± 30.06	83.50 ± 81.47	0.395
**Homoarginine (μmol/L)**	2.61 ± 1.59	2.71 ± 1.13	0.677
**Citrulline (μmol/L)**	9.34 ± 8.10	8.59 ± 6.84	0.578
**ADMA (μmol/L)**	0.19 ± 0.05	0.26 ± 0.20	**0.022**
**SDMA (μmol/L)**	0.23 ± 0.07	0.27 ± 0.08	**0.008**
**Arginine/ADMA ratio**	401.61 ± 177.40	392.59 ± 408.09	0.881
**SDMA/ADMA ratio**	1.27 ± 0.42	1.26 ± 0.51	0.888
**Hb (g/dL) (13.5 – 17.2)**	13.69 ± 0.65	13.36 ± 0.82	**0.016**
**HCT (%) (39.5–50.5)**	43.43 ± 2.03	42.88 ± 2.29	0.170
**Platelet count(10** **^3^** **/μL) (150 – 400)**	228.83 ± 37.11	227.17 ± 40.06	0.813
**MCV (fL) (80–99)**	89.10 ± 2.63	88.09 ± 2.96	0.052
**MCH (pg) (27–33.5)**	29.95 ± 1.25	29.68 ± 1.28	0.240
**MCHC (g/dL) (31.5–36)**	33.35 ± 0.80	33.42 ± 0.92	0.660
**RDW (%) (11.8–14.5)**	14.42 ± 0.66	14.50 ± 0.69	0.512
**Ferritin (μg/L) (22–322)**	54.47 ± 25.26	47.00 ± 25.50	0.107
**Serum iron (μg/dL) (65–175)**	93.11 ± 20.98	80.86 ± 25.56	**0.005**
**Transferrin (mg/dL) (200–360)**	357.05 ± 79.76	367.13 ± 97.90	0.540
**TIBC (μg/dL) (240–450)**	339.68 ± 72.10	331.25 ± 73.67	0.525

Data are presented as mean ± SD. Statistical significance values were obtained using independent t-tests comparing the short-term (1–5 years) and long-term (≥ 6 years) groups. Bold values indicate p < 0.05.

**Table 3:** Correlations between blood Pb levels and selected biomarkers.

Variable	r	p-value
**SDMA**	0.224	**<0.001**
**Citrulline**	−0.460	**<0.001**
**Hb**	−0.476	**<0.001**
**Ferritin**	−0.503	**<0.001**
**MCV**	−0.465	**<0.001**
**MCH**	−0.532	**<0.001**
**Transferrin**	0.370	**<0.001**
**TIBC**	0.355	**<0.001**

Pearson correlation coefficients (r) are presented. All reported correlations were statistically significant (p < 0.05).

**Table 4:** Multivariable linear regression analyses for selected biomarkers.

Dependent Variable (n = 250)	Predictor	B	SE	β	t	95% CI	p	VIF
**SDMA (μmol/L)**	Age	0.000	0.000	−0.035	−0.559	−0.001 to 0.001	0.577	1.05
	Pb	0.001	0.000	0.220	3.514	**0.000 to 0.001**	**0.001**	1.08
	BMI	0.001	0.001	0.055	0.880	−0.001 to 0.003	0.379	1.06
**Model fit**	**R** **^2^** ** = 0.054, F = 4.669, p = 0.003**							
**Hb (g/dL)**	Age	0.012	0.007	0.097	1.715	−0.002 to 0.026	0.088	1.05
	Pb	−0.025	0.003	−0.464	−8.257	**−0.031 to −0.019**	**<0.001**	1.08
	BMI	−0.014	0.018	−0.043	−0.763	−0.050 to 0.022	0.446	1.06
**Model fit**	**R** **^2^** ** = 0.237, F = 25.424, p < 0.001**							
**Citrulline (μmol/L)**	Age	0.092	0.092	0.057	0.997	−0.090 to 0.273	0.320	1.05
	Pb	−0.313	0.039	−0.453	−7.943	**−0.391 to −0.236**	**<0.001**	1.08
	BMI	0.032	0.237	0.008	0.135	−0.435 to 0.499	0.892	1.06
**Model fit**	**R** **^2^** ** = 0.215, F = 22.456, p < 0.001**							
**Arginine (μmol/L)**	Age	0.129	0.349	0.024	0.370	−0.558 to 0.815	0.712	1.05
	Pb	−0.254	0.149	−0.108	−1.700	−0.548 to 0.040	0.090	1.08
	BMI	1.055	0.897	0.075	1.176	−0.712 to 2.822	0.241	1.06
**Model fit**	**R** **^2^** ** = 0.019, F = 1.602, p = 0.190**							
**Platelet (10** **^3^** **/μL)**	Age	−0.113	0.263	−0.028	−0.431	−0.632 to 0.405	0.667	1.05
	Pb	0.152	0.113	0.086	1.343	−0.071 to 0.374	0.181	1.08
	BMI	−0.270	0.678	−0.025	−0.398	−1.605 to 1.066	0.691	1.06
**Model fit**	**R** **^2^** ** = 0.010, F = 0.798, p = 0.496**							
**Ferritin (μg/L)**	Age	0.154	0.197	0.044	0.781	−0.234 to 0.542	0.436	1.05
	Pb	−0.758	0.084	−0.498	−8.973	**−0.924 to −0.591**	**<0.001**	1.08
	BMI	−0.374	0.507	−0.041	−0.737	−1.373 to 0.625	0.462	1.06
**Model fit**	**R** **^2^** ** = 0.256, F = 28.218, p < 0.001**							

Values represent unstandardized regression coefficients (B), standard errors (SE), standardized coefficients (β), t statistics, 95% confidence intervals (CI), p values, and variance inflation factors (VIF). All models were adjusted for age and body mass index. Bold values indicate p < 0.05.
